# Personnel well-being and potentially traumatic COVID-19 pandemic related events (PTES) in the hus helsinki university hospital – baseline results

**DOI:** 10.1192/j.eurpsy.2021.741

**Published:** 2021-08-13

**Authors:** T. Laukkala, H. Haravuori, K. Tuisku, K. Junttila, T. Haapa, A. Kujala, E. Pukkala, J. Suvisaari, T. Rosenström, P. Jylhä

**Affiliations:** 1 Hy, HUS, Helsinki, Finland; 2 Psychiatry, HUS, HUS, Finland; 3 Faculty Of Social Sciences, Tampere University, Tampere, Finland; 4 Mental Health Unit, Finnish Institute for Health and Welfare, Helsinki, Finland; 5 Psychology And Logopedics, Helsinki University, Helsinki, Finland; 6 Acute And Consultation Psychiatry, HUS, Helsinki, Finland; 7 Psychiatry, HUS, Helsinki, Finland

## Abstract

**Introduction:**

A majority of the Finnish COVID-19 pandemic patients have been cared for in the HUS Helsinki University Hospital since March 2020.

**Objectives:**

June 2020 baseline results of an ongoing prospective cohort study are reported.

**Methods:**

An electronic survey was created to assess potentially traumatic COVID-19 pandemic related events (PTEs) of the HUS personnel.

**Results:**

The survey was sent to 25494 HUS employees, and 4804 (19%) answered. Out of the respondents, 62% were nursing staff, 9% medical doctors, and the rest special employees or other personnel. Mean age was 44 years, 88% were female. PTEs were more common in the personnel directly caring for COVID-19 patients than other personnel (p< 0.001). PTEs predicted psychological distress among all personnel (OR 5.05; 95%CI 4.26–6.00). Table. Potentially traumatic events (PTEs) among HUS personnel, June 2020. One respondent may have one or more PTEs.

**Table:** 

In direct care of COVID-19 patients	PTE1^1^	PTE2^2^	PTE3^3^	PTE4^4^	Respondents
Yes (N; %)	325 (26.6%)	358 (29.3%)	46 (3.8%)	9 (0.7%)	532
No (N; %)	281 (8.2%)	574 (16.6%)	88 (2.5%)	30 (0.9%)	760

^1^Has your work with COVID-19 patients or suspected patients included exceptionally disturbing or distressing assignments? ^2^Have you had strong anxiety due to your own or close one’s risk of contracting serious illness for your work with COVID-19 patients or suspected patients? ^3^Have you or your close one contracted a hospital care requiring serious COVID-19? ^4^Has a close one to you died of COVID-19?

**Conclusions:**

Our data highlight the need to ensure psychosocial support services to HUS personnel with PTEs.

**Conflict of interest:**

No significant relationships.

